# Effect of vibration vs non-vibration foam rolling techniques on flexibility, dynamic balance and perceived joint stability after fatigue

**DOI:** 10.7717/peerj.8000

**Published:** 2019-11-26

**Authors:** Ana María de Benito, Raúl Valldecabres, Diego Ceca, Jim Richards, Joaquín Barrachina Igual, Ana Pablos

**Affiliations:** 1 Physical Activity and Sports Sciences Faculty, Universidad Católica de Valencia San Vicente Mártir, Torrent, Valencia, Spain; 2 Doctorate School, Universidad Católica de Valencia San Vicente Mártir, Valencia, Spain; 3 Department of Education, Universidad Internacional de Valencia, Valencia, Spain; 4 Allied Health Research Unit, University of Central Lancashire, Preston, UK

**Keywords:** Recovery, ROM, Vibration foam roller, Ankle dorsiflexion, Foam roller

## Abstract

**Background:**

Foam roller and vibration techniques are currently used to assist in recovery after fatigue. The main purpose of this study was to determine the effects of the use of a foam roller with and without vibration on dynamic balance, ankle dorsiflexion, hamstring and lumbar spine flexibility and perceived knee and ankle stability after an induced fatigue protocol.

**Methods:**

A total of 24 healthy recreationally active participants (17 males and seven females) were recruited to a randomized cross over trial consisting of; no treatment (NT), foam roller treatment (FR) and vibration foam roller treatment (VFR). The assessments included; the Sit & Reach test, *Y* balance test and post-treatment perceived knee and ankle stability. Measurements were taken after a standardized warm up (baseline) and repeated following an exercise-induced muscle fatigue protocol consisting of repeated lunges until volitional fatigue. The three treatment conditions were assessed on three separate days in a randomized order. A 3 × 3 repeated measures ANOVA was used to investigate differences between the three treatments over the three time points and a one factor repeated measures ANOVA was used to determine any differences between treatments using the Global Rate of Change scale when considering perceived stability.

**Results:**

FR and VFR conditions both showed a greater ankle dorsiflexion range of motion (ROM) (*p* < 0.001), greater posteromedial and posterolateral reach distances (*p* < 0.001) and a better knee and ankle perceived stability (*p* < 0.001) when compared to the NT condition. A trend toward significance was observed in the hamstring and lumbar spine flexibility (*p* = 0.074) in both treatment conditions when compared to the NT condition. However, no differences were seen between the FR and VFR conditions.

**Conclusions:**

Both FR conditions seem to assist in exercise-induced muscle fatigue recovery with improvements in ROM, balance and perceived stability.

## Introduction

It is known that high-intensity exercise can induce acute and chronic fatigue, potentially causing performance impairment and/or injuries (Ekstrand, Waldén & Hägglund, 2004; Dupont et al., 2010). The underlying mechanisms of exercise-induced fatigue are understood to be a multifaceted process involving peripheral to central factors (Jo et al., 2018). Ratel, Duché & Williams (2006) indicated that about 80% of fatigue after high-intensity dynamic exercise could be accounted for from a peripheral origin. This can include physical signs such as disrupted sarcomeres and damage to components of the excitation-contraction coupling system. During these events, the temporary symptoms include stretch-reflex sensitivity, muscle joint stiffness regulation and an increase in perceived fatigue (Eston, Byrne & Twist, 2003). Ribeiro, Mota & Oliveira (2007) add that muscle fatigue may impair the proprioceptive and kinesthetic properties of joints by increasing the threshold of muscle spindle discharge, disrupting afferent feedback and subsequently altering conscious joint awareness. Therefore, fatigue can cause performance impairment and increase injury risk, so to carry out athletic activity safely, it is important to develop different physical performance components which include balance and proprioception (Manske & Davies, 2003; Myer et al., 2011; Manske & Reiman, 2013) and joint stability (Lee et al., 2018).

Balance testing has been shown to be an effective method to assess the risk of injuries and offers an integral part of assessment of athletic ability, alongside power and agility (Gribble & Hertel, 2003; McLeod et al., 2009; Hrysomallis, 2011). It has been suggested that as power and agility improve, balance ability must adapt to be able to control these functional movement patterns, resulting in improved performance (Manske & Reiman, 2013).

Joint flexibility is also an important component to consider. Joint range of motion (ROM), as well as the resistance to stretching (i.e., muscle and tendon stiffness) are important physical characteristics that influence the capacity to perform athletic tasks and can be associated with muscle strain injury risk, especially in high-intensity activities that involve many stretch-shortening cycles (Witvrouw et al., 2004; Watsford et al., 2010).

Therefore, practical strategies are needed to relieve acute exercise-induced fatigue and maintain muscular performance capacity during exercise or competitive sports.

The use of foam roller techniques has become one of the most widely utilized modalities to decrease the risk of injury by offering improvements in the recovery cycle (Jo et al., 2018). This technique requires individuals to use their own body mass to apply pressure to the soft tissue via a foam roller (MacDonald et al., 2014). The effects of foam roller techniques on recovery after an induced fatigue protocol have been reported, these include improvements in perceived fatigue, a reduction of muscle pain and an increase in blood flow (Cheatham et al., 2015; Hotfiel et al., 2017; Romero-Moraleda et al., 2019). It has been theorized that foam rollers may potentiate analgesic effects and muscular recovery by mediating pain-modulatory systems (e.g., nociceptor and mechanoreceptor sensitivity and diffuse noxious inhibitory control) (Cavanaugh et al., 2017; Jo et al., 2018). In addition, it has been reported that the pressure exerted using foam roller techniques is able to reduce the arterial stiffness and produce improvements in vascular endothelium function (Okamoto, Masuhara & Ikuta, 2014). Moreover, psychophysiological responses may also include enhanced perceptions of well-being and recovery due to the increase of plasma endorphins, a decreased arousal level and an activation of the parasympathetic response (Weerapong, Hume & Kolt, 2005; Phillips et al., 2018).

The use of vibration therapy has become a popular recovery method over the last decade and has been reported to reduce muscle soreness and fatigue (Kang et al., 2017; Iodice, Ripari & Pezzulo, 2019) and can easily be incorporated in injury prevention programs for athletes. However, little is known about the effectiveness of the combination of foam roller with vibration on recovery (Cheatham, Stull & Kolber, 2017; García-Gutiérrez et al., 2018; Romero-Moraleda et al., 2019). Some authors have reported greater improvements when considering knee and hip range of movement (Cheatham, Stull & Kolber, 2017; Han, Lee & Lee, 2017), indicating a reduction in muscle tightness. In addition, Cheatham, Stull & Kolber (2017), Han, Lee & Lee (2017) and Romero-Moraleda et al. (2019) all reported lower perceived pain when using vibration. However, García-Gutiérrez et al. (2018) and Romero-Moraleda et al. (2019) showed that foam roller treatment (FR) produced increases in knee and ankle ROM with respect to a control group regardless of the use of vibration. Moreover, no studies have thoroughly examined the effects of foam roller with vibration on recovery over dynamic balance, stability and muscle-tendon stiffness. Lee et al. (2018) found that vibration may be helpful in improving dynamic balance through the effective improvement of muscle strength of the quadriceps muscles when used as part of a warm-up routine, but to date the application and effects in a fatigue state has not been reported.

Therefore, the purpose of this study was to assess the effects of foam roller and foam roller with vibration on ankle dorsiflexion ROM, hamstring and lumbar spine flexibility, dynamic balance and perceived knee and ankle stability after an induced fatigue protocol.

## Materials and Methods

### Study design

This study was a randomized cross over trial carried out between May 2018 and July 2018 in the Movement Analysis Laboratory at the Universidad Católica de Valencia San Vicente Mártir (Torrente, Spain). Dorsiflexion ROM, hamstring and lumbar flexibility and dynamic balance were measured at baseline, post-fatigue and post-treatment, on three separate days for three conditions; no treatment (NT), foam roller treatment (FR) and vibration foam roller treatment (VFR) in healthy participants. Moreover, post-treatment perceived knee and ankle stability were measured.

### Participants

An a priori power analysis (G*Power3) of predicted changes in ROM following self-massage, with α = 0.05 and 1 − β = 0.80 indicated that a sample size of 24 participants was required. Therefore, data were recorded from the left and right sides from 24 healthy recreationally active participants (17 males and seven females). Eligibility criteria were; 18–28 years old, a classification of “vigorous physical activity” using the International Physical Activity Questionnaire (Craig et al., 2003), free from musculoskeletal or traumatic injuries, neurological or balance disorders or medical conditions that could limit physical activity.

This study was conducted according to the guidelines laid down in the Declaration of Helsinki and all procedures involving human subjects were approved by the UCV Ethics Committee (Ref. UCV2017-2018-30). All participants were provided with a detailed explanation of the study procedures and participants gave written informed consent prior to data collection.

Body weight (BW) was measured to the nearest 0.01 kg and height was measured to the nearest 0.1 cm using a Seca 200 scale with an attached stadiometer (Seca, Hamburg, Germany). Body mass index was calculated by BW (kg) divided by height squared (m^2^). The protocols followed were those established by the International Society for Advancement of Kinanthropometry (ISAK) (Stewart & Marfell-Jones, 2006) and all data were recorded by a qualified professional (ISAK Level I).

### Data collection

A Leg Motion system (Check your Motion^®^, Albacete, Spain) was used to evaluate the ankle dorsiflexion ROM. Participants were in a standing position with their hands on their hips with the test foot on the measurement scale. The contralateral foot was positioned to the side of the platform. While maintaining this position, participants were instructed to perform a lunge forward. Participants were asked to maintain contact between the anterior knee and the perpendicular metal bar as it was moved forwards without lifting their heel and this position had to be held for 3 s (Calatayud et al., 2015). Three trials were performed for the dominant and non-dominant sides and the mean value of each side was recorded. Previous research indicates every one cm away from the metal bar is equivalent to approximately 3.6° of ankle/subtalar dorsiflexion (Bennell et al., 1998). Croxford, Jones & Barker (1998) suggested that clinicians using goniometry should only assume that a real clinical change in ankle dorsiflexion has occurred when there has been a ROM change bigger than 5°, but Winter (1992) collected gait laboratory data demonstrating that foot clearance is sensitive to angular changes as small as 2.07° at the ankle.

The *Y* balance test was used to assess dynamic balance (Plisky et al., 2006, 2009). During the test, all participants stood on the center of the foot plate with a single leg with the most distal aspect of the shoe at the starting line. Participants were asked to maintain single leg stance and reach with the free limb as far as possible in three directions; posterolateral, posteromedial and anterior. They completed three consecutive trials for each direction. The reach distance data were registered and normalized to leg length, which was measured with a tape measure from the anterior superior iliac spine to the center of the medial malleolus (Terry et al., 2005). Aminaka & Gribble (2005) proposed that a minimum clinically important change in dynamic stability must be larger than 5% of limb length, which equates to approximately a five cm reach difference and can influence most sports’ tasks.

The Sit and Reach (S&R) test was used to assess hamstring and lumbar spine flexibility (Grieve et al., 2015) using an S&R box. Participants were seated on the floor with the heels/soles of their feet flat against the box. In addition, subjects were instructed to reach forward as far as possible preserving the lower limb position (fully extended knees), with their fingertips pushing the measuring gage and holding the maximal reach for 2 s. S&R test was carried out three times and the maximum length reached was recorded. It has been proposed that a real change in hamstring flexibility must be larger than 6.72% (Ayala et al., 2012).

In addition to these assessments, participants were asked to evaluate their knee and ankle perceived stability using a five-point Global rate of change (GROC) scale: (1) quite a bit worse, (2) slightly worse, (3) about the same, (4) slightly better, (5) quite a bit better. The minimum clinically important change using an 11-point GRC scale has been previously reported as a score of 2 (Kamper, Maher & Mackay, 2009) so a change of 1 could be used in a five-point scale.

### Procedure

Participants were required to visit the laboratory on four occasions. The first visit was used as a training session. Participants received verbal instruction and a visual demonstration of the tests. Subsequently, they were allowed to familiarize themselves with the protocol by performing as many repetitions as they needed to feel comfortable with the tests to reduce any learning effects (Gribble, Hertel & Plisky, 2012). Anthropometric measurements were taken and the order of the conditions for the next three visits was randomized. Test sessions were carried out on three separate days, with 7 days between each treatment condition, giving participants enough time to rest. Participants were encouraged to avoid performing high intensity physical activity 24 h before the test sessions.

At the beginning of each test session, participants performed a standardized warm-up of 10 min of active stretching and 5 min of cycling. Subsequently, participants performed the protocol in a previously allocated randomized order. After the warm up, baseline measurements were taken. An exercise-induced muscle fatigue protocol was then performed (Pincivero et al., 2000), which consisted of repeating lunges until volitional maximum fatigue was reached. Lunge distance for each participant was determined as a proportion of the participants’ leg length. A metronome was used to standardized lunge rate which was set to 30 repetitions per minute. The fatigued state was considered to have been reached when participants could no longer keep the rhythm of the lunge task for two consecutive repetitions. Immediately after the fatigue protocol, participants performed the three clinical tests in the same randomized order as baseline. The FR conditions (FR and VFR) were then applied and for NT participants were asked to remain seated for the same length of time as the treatment conditions. The treatment conditions consisted of a self-administered myofascial release technique comprising of two rounds of a 60 s application with 30 s rest on each muscle group (Vigotsky et al., 2015). The foam roller was applied bilaterally on the quadriceps and hamstrings at a slow pace (1 s down and 1 s up) using a metronome. In addition, the maximum possible pressure was applied between the origin and insertion of the muscles. Immediately after the treatment conditions the clinical tests were repeated in the same order.

The foam roller device used was a Blackroll PRO (Bottighofen, Switzerland) with a density of 2.3 kg·m^−3^. The vibration was applied at 30 Hz which has been reported as the optimal frequency range to effect the musculoskeletal system (Slatkovska et al., 2010; Luo et al., 2017).

### Statistical analysis

Descriptive statistics were used to explore the subject characteristics and the results of ankle dorsiflexion ROM, *Y* balance-test and S&R test. A frequency analysis was also used to determine the perceived stability after treatment. All data were examined for normality using the Kolmogorov–Smirnov test and found suitable for parametric testing. No significant differences between sides were seen, therefore giving a sample size of 48 lower limbs. A 3 × 3 repeated measures ANOVA was used to investigate if differences exist between the treatment conditions (NT, FR, VFR) and time points (T1, T2, T3). Least significant difference (LSD) pairwise comparisons were used to investigate the main effects of group and time. One factor repeated measures ANOVAs with LSD pairwise comparisons were used to check differences between treatments in perceived joint stability. All analyses were performed using IBM SPSS Statistics v23.0 (SPSS Inc., Chicago, IL, USA) software. Statistical significance was set at *p* < 0.05.

## Results

**Figure 1:**
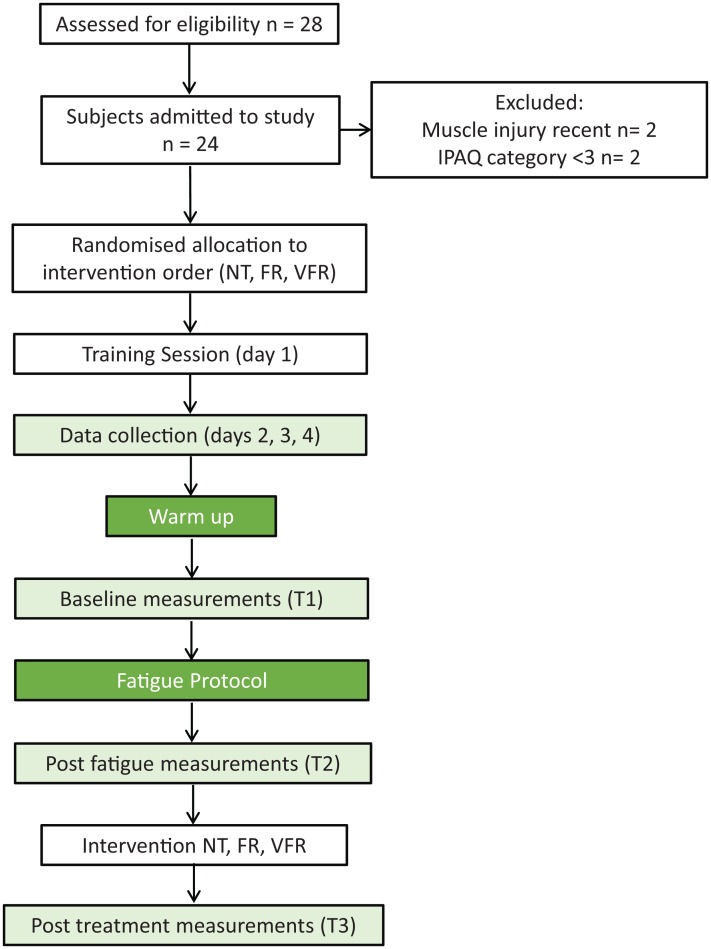
Recruitment and testing flow diagram. Note: T1, Baseline; T2, post-fatigue; T3, post-treatment; NT, no treatment; FR, foam roller treatment; VFR, vibration foam roller treatment.

The consort diagram for recruitment and testing is presented in Fig. 1 and the characteristics of the participants are shown in Table 1. The mean and standard deviation and main effects for the different variables are reported in Table 2. An interaction between treatment and time was seen on the ankle dorsiflexion ROM and posteromedial and posterolateral reach measures of the *Y* balance test. There was also tendency towards an interaction for the anterior reach test within the *Y* balance test and S&R.

**Table 1:** Characteristics of the subjects.

Parameters	Men (*n* = 17)	Women (*n* = 7)	Total (*n* = 24)
	Mean ± SD	Mean ± SD	Mean ± SD
Age, years	22.50 ± 0.38	20.43 ± 0.51	21.78 ± 2.41
Weight, kg	73.43 ± 1.41	56.64 ± 1.67	69.08 ± 11.10
Height, cm	177.03 ± 0.72	160.86 ± 1.78	172.84 ± 8.79
BMI, kg/m^2^	23.41 ± 0.57	21.89 ± 0.82	23.01 ± 2.52

**Note:**

BMI = Body mass index.

**Table 2:** Descriptive and inferential results from 3 (treatment) × 3 (time) ANOVA.

Treatment/time		Dorsiflexion, cm		Anterior reach, m		Posteromedial reach, m		Posterolateral reach, m		S&R, cm	
		*M*	SD	*M*	SD	*M*	SD	*M*	SD	*M*	SD
NT	T1	10.70	2.3	0.61	0.1	1.08	0.1	1.04	0.1	2.33	10.4
	T2	10.56	2.4	0.59	0.0	1.06	0.1	1.01	0.1	4.11	8.9
	T3	10.25	2.5	0.59	0.0	1.07	0.1	1.03	0.1	4.03	8.8
FR	T1	10.63	2.6	0.60	0.0	1.09	0.1	1.04	0.1	2.92	9.5
	T2	10.60	2.5	0.58	0.0	1.06	0.1	1.02	0.1	3.94	8.8
	T3	10.98	2.4	0.60	0.0	1.09	0.1	1.06	0.1	5.15	8.8
VFR	T1	10.68	2.5	0.61	0.1	1.07	0.1	1.03	0.1	3.41	8.6
	T2	10.83	2.4	0.59	0.1	1.07	0.1	1.03	0.1	3.97	8.9
	T3	11.04	2.4	0.61	0.1	1.09	0.1	1.06	0.1	5.13	9.0
**RM ANOVA**		***p***	**η^2^*_p_***	***p***	**η^2^*_p_***	***p***	**η^2^*_p_***	***p***	**η^2^*_p_***	***p***	**η^2^*_p_***
Group		0.142	0.08	0.014	0.18	0.238	0.06	0.074	0.11	0.360	0.09
Time		0.432	0.04	<0.001	0.49	<0.001	0.50	<0.001	0.68	<0.001	0.58
Intera.		<0.001	0.44	0.083	0.17	<0.001	0.44	0.001	0.34	0.074	0.35

**Note:**

S&R, Sit & Reach Test; NT, no treatment; FR, foam roller treatment; VFR, vibration foam roller treatment; T1, Baseline; T2, post-fatigue; T3, post-treatment; Intera., Interaction.

**Figure 2:**
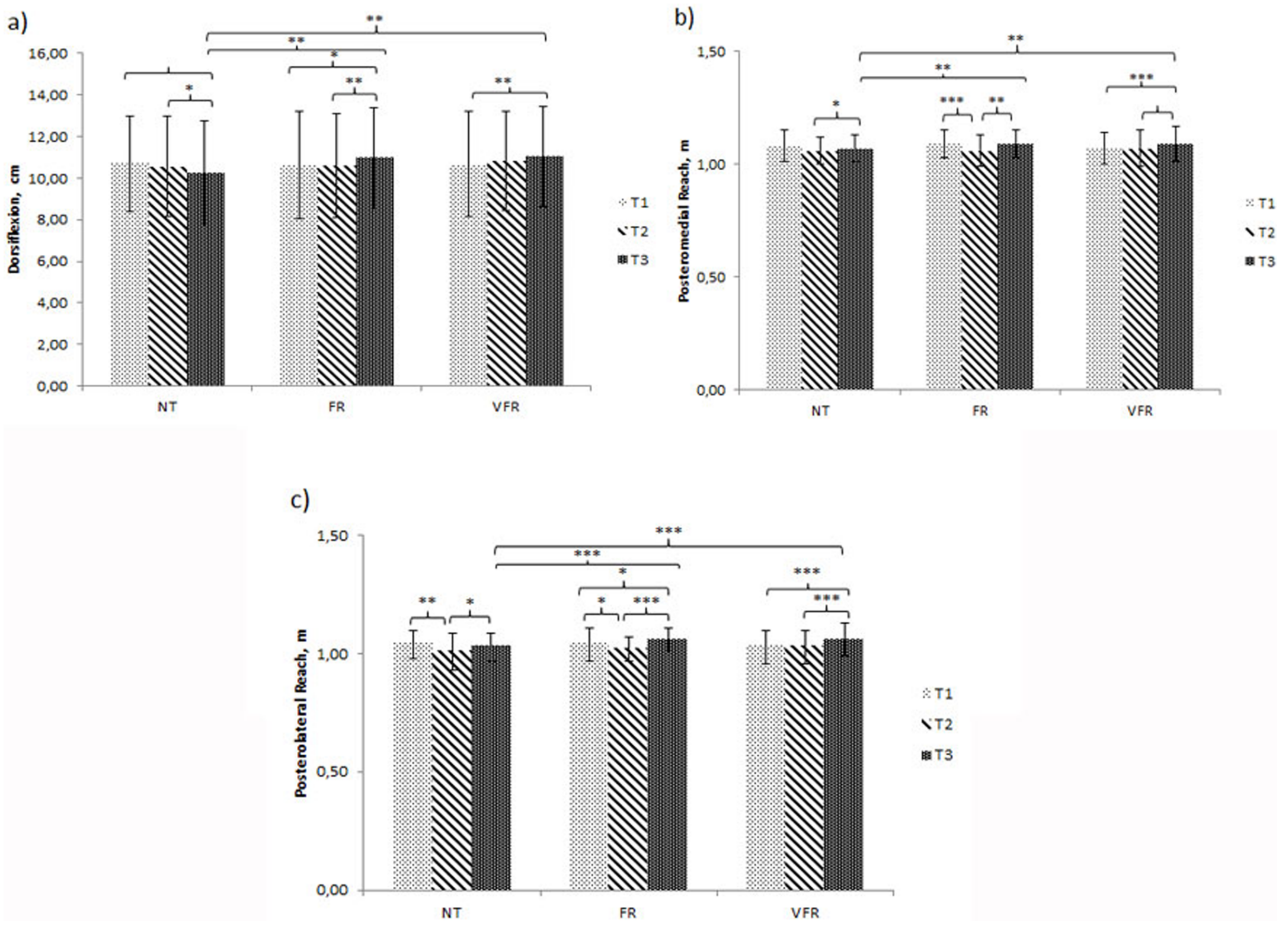
Results for time × treatment ANOVA showing the simple effects for the significant variables. (A) Ankle dorsiflexion. (B) Dynamic balance, posteromedial reach. (C) Dynamic balance, posterolateral reach. Note: T1, Baseline; T2, post-fatigue; T3, post-treatment; NT, no treatment; FR, foam roller treatment; VFR, vibration foam roller treatment. Significance level: ***p < 0.001; **p < 0.01; *p < 0.05.

Significant improvements were seen between the time points for both FR and VFR treatments and between both treatments and NT. However, no significant differences were seen between the FR and VFR treatment conditions (Fig. 2).

**Figure 3:**
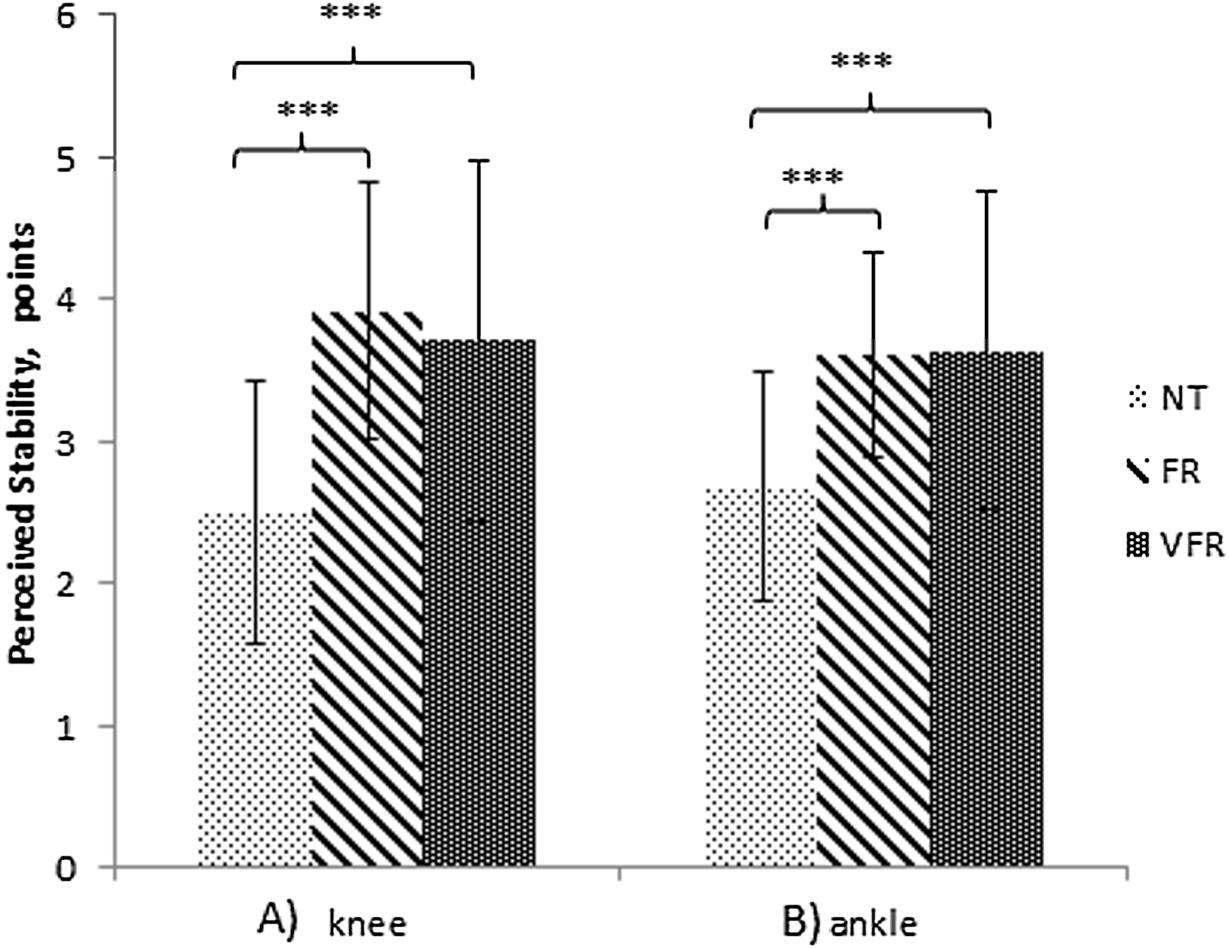
Simple effects for the joints perceived stability. (A) Knee perceived stability. (B) Ankle perceived stability. Note: NT, no treatment; FR, foam roller treatment; VFR, vibration foam roller treatment. Significance level: ***p < 0.001.

The GROC for knee and ankle joint stability is presented in Table 3. The interaction effect can be seen descriptively in Fig. 3. Results show a greater perceived joint stability after both treatments, regardless of vibration. The percentage of participants who perceived improvement or worsening after treatment can be seen in Table 4.

**Table 3:** Descriptive and inferential results from one factor repeated measures ANOVA for perceived joint stability.

Variable	Treatment	Mean	SD	*F*	*p*	η^2^*_p_*
Perceived knee stability	NT	2.50	0.9	26.98	<0.001	0.42
	FR	3.92	0.9			
	VFR	3.71	1.27			
Perceived ankle stability	NT	2.68	0.8	15.01	<0.001	0.29
	FR	3.61	0.7			
	VFR	3.63	1.1			

**Note:**

NT, no treatment; FR, foam roller treatment; VFR, vibration foam roller treatment.

**Table 4:** Results from frequency analysis for perceived joint stability.

Treatment	Perceived knee stability			Perceived ankle stability		
	Quite a bit/slightly worse	About the same	Slightly/quite a bit better	Quite a bit/slightly worse	About the same	Slightly/quite a bit better
NT (%)	58.3	16.7	25.0	29.2	58.3	12.5
FR (%)	7.7	25.0	67.3	0	46.2	53.8
VFR (%)	13.5	3.8	82.7	0	36.5	63.5

**Note:**

NT, no treatment; FR, foam roller treatment; VFR, vibration foam roller treatment.

## Discussion

This study compared the effectiveness of a self-administered myofascial release technique using a foam roller with and without vibration after an induced fatigue protocol. Significant improvements in ankle dorsiflexion ROM (*p* < 0.001) and *Y* balance in the posteromedial reach (*p* < 0.001) and posterolateral reach (*p* < 0.001) were seen and an increase in perceived ankle stability (*p* < 0.001) and knee stability (*p* < 0.001) was seen between the time points in both FR and VFR conditions compared to the NT condition. In addition, there was also a tendency toward an improvement in hamstring and lumbar spine flexibility in both treatments.

When considering the ankle ROM, it has been previously shown that there is an increase of muscle stiffness after an induced fatigue protocol (Lacourpaille et al., 2014). Applying foam roller post fatigue has been shown to increase joint flexibility and ROM by reducing muscle stiffness (Schroeder & Best, 2015; Cheatham et al., 2015; Romero-Moraleda et al., 2019). This is supported by the findings in this current study which showed a greater ankle dorsiflexion ROM, with significant increases in the FR (+3.3%) and in the VFR (+3.4%) compared to NT (−4.2%). Both treatment conditions obtained similar short-term outcomes, which is supported by Romero-Moraleda et al. (2019) and Halperin et al. (2014) using similar protocols. In this current study, the changes due to the FR treatments could be considered as clinically important, as the change seen was greater than 2.07° compared to NT (Winter, 1992). In addition, a similar magnitude of change was seen when using VFR, with a change of 1.9° compared to NT. There are many theories which attempt to explain the effects obtained during myofascial release which can occur during foam roller interventions (Dębski, Białas & Gnat, 2019). These effects include the elimination of symptoms known as fascial restrictions and adhesions (Patel, Vyas & Sheth, 2016; Morton et al., 2016), an increase in stretch tolerance (Weppler & Magnusson, 2010) and a modulation of pain induced analgesia (Aboodarda, Spence & Button, 2015; Cavanaugh et al., 2017; Romero-Moraleda et al., 2019). It has also been hypothesized that vibration could further improve ROM as a result of an increase in temperature and blood flow (Veqar & Imtiyaz, 2014), muscle activity and metabolic response (Pamukoff, Ryan & Blackburn, 2014). However, the findings of this current study showed similar effects in both treatments which is supported by previous studies (Cheatham, Stull & Kolber, 2017; García-Gutiérrez et al., 2018), even when what is considered as an optimal frequency (30 Hz) was selected (Luo et al., 2017). It is plausible that the force applied through the foam roller may have nullified the cumulative effect of vibration, so a higher vibration frequency or higher amplitude may be required to ensure the efficacy of vibration as it has been suggested by García-Gutiérrez et al. (2018).

For the S&R test, which specifically measures hamstrings and lumbar spine flexibility, no significant differences (*p* = 0.074) were found, but a trend toward significance was observed for both treatment conditions (FR +30.7%; VFR +29.2%). These results are higher than the 6.72% threshold for an important difference, therefore they could be considered as clinically important according to Ayala et al. (2012), despite not being statistically significant. In addition, these findings are similar to those reported by Schroeder et al. (2019) who showed an increase of 30.26% when a foam roller protocol was applied on paraspinal and lateral back muscles in healthy young females. Although several authors show flexibility improvements after foam roller application in a fatigued state, considerable variability in the results obtained on lumbar spine flexibility improvements have been reported (Grieve et al., 2015; Jung et al., 2017; Rey et al., 2019; Schroeder et al., 2019). Protocol variations could be responsible for the differences in findings which include pressure exerted by the roller, duration of intervention and speed of application (Dębski, Białas & Gnat, 2019).

Significant improvements in both treatment (FR and VFR) conditions were seen in the posteromedial (*p* < 0.001) and posterolateral (*p* < 0.001) directions with a tendency toward a significant difference in the anterior direction (*p* = 0.083) during the *Y* balance test. One explanation could be that the use of foam roller techniques after fatigue accelerates restoration process of the nervous pathways to peripheral factors and the soft tissue (Wiewelhove et al., 2019), which may improve knee stability due to a decrease in stiffness, which may result in a reduction in injury risk (Padua et al., 2006). Padua et al. (2006) stated that muscle recruitment strategy is modified when participants are in a fatigued state. This change supports that reducing voluntary muscle activation, due to swelling or stiffness, can also contribute to a reduction in muscular function (Byrne, Twist & Eston, 2004).

Another explanation is that proprioception is a critical factor that can increase the susceptibility to joint sprain injuries as it provides immediate feedback to joint stability when joint proprioception is disturbed (Lee et al., 2018). However, our results are different from those obtained by Lee et al. (2018), who only found a significantly improved dynamic balance after vibration roller application. Protocol differences may be responsible for this difference in findings as Lee et al. (2018) only applied one round of a 30 s intervention, while this current study applied two rounds of 60 s each after an induced fatigue protocol. Therefore, further research is required to explore the application dosage of foam roller after induced fatigue protocols and its potential in reducing the risk of injury when in a fatigued state.

Dynamic balance outcomes are related to those obtained from subjects’ knee and ankle perceived stability. It has been shown that foam rolling treatments, regardless of vibration, improve the perception of stability in both the ankle and knee joints when compared with NT. The percentage of participants who perceived an improvement in knee stability after treatment was between 67.3% and 82.7% for FR and VFR, respectively, with only 25% of participants showing an improvement after NT through natural recovery. Similar changes were seen when ankle perceived stability was considered with FR showing a 53.8% improvement and VFR showing a 63.5% improvement in contrast to NT with only a 12.5% improvement. These findings could be related to an improvement of joint proprioception due to sensory input into the muscle mechanoreceptors (Skinner et al., 1986; Bartlett & Warren, 2002). In addition, according to Bordoni, Lintonbon & Morabito (2018), enhancement of perceived stability is not only seen in the joints directly associated with the muscles treated, but also in the joints along the fascial chain.

This study has several limitations. Firstly, this investigation was performed only in healthy and active participants, which limits the generalizability of these results to other populations. Secondly, the vibrating roller was used at only one frequency (30 Hz). Although this is reported as the optimal frequency range to affect the musculoskeletal system (Luo et al., 2017), other frequencies or wave widths may have generated different outcomes. Further research is required to determine the optimal applied force when vibration is combined with foam rolling as suggested by García-Gutiérrez et al. (2018). This could be due to the attenuation produced by the foam in the vibration wave width. Thirdly, measurements were only performed immediately after the interventions, so further work needs to consider how this compares to a longer period of natural recovery.

## Conclusions

Foam roller treatments (FR and VFR) applied after an induced fatigue protocol significantly increased ankle dorsiflexion ROM, dynamic balance and perception of knee and ankle stability. There was also an improvement in hamstring and lumbar spine flexibility, however no differences were seen when vibration was added. The use of foam roller appears to assist recovery after fatigue which may be considered as beneficial practice for athletic professionals when trying to achieve a quicker recovery.

## Supplemental Information

10.7717/peerj.8000/supp-1 Supplemental Information 1 Raw data.

10.7717/peerj.8000/supp-2 Supplemental Information 2 Codebook.

## Additional Information and Declarations

### Data Availability

The following information was supplied regarding data availability: The raw measurements are available in File S1.
