# Supplementary material for: Effect of vibration vs non-vibration foam rolling techniques on flexibility, dynamic balance and perceived joint stability after fatigue
Source: PeerJ. 2019 Nov 26;7:e8000. doi: 10.7717/peerj.8000 (PMC6883953; doi:10.7717/peerj.8000)
Supplement: Supplemental Information 2 [file peerj-07-8000-s002.pdf]

Variable	code
Treatment	0: No tratment
	1: Foam Roller treatment
	2: Vibration foam roller treatment
Sex	1: Men
	2: Women
p-stability knee	1: quite a bit worse
	2: slightly worse
	3: about the same
	4: slightly better
	5: quite a bit better
p-stability ankle	1: quite a bit worse
	2: slightly worse
	3: about the same
	4: slightly better
	5: quite a bit better
